# Chemical Composition and Antifungal, Anti-Inflammatory, Antiviral, and Larvicidal Activities of the Essential Oils of *Zanthoxylum acanthopodium* DC. from China and Myanmar

**DOI:** 10.3390/molecules27165243

**Published:** 2022-08-17

**Authors:** Jingjing Yang, Xingzhen Song, Huabin Hu, Wu Zhong, Ruiyuan Cao, Youkai Xu, Ren Li

**Affiliations:** 1 School of Pharmaceutical Sciences, Hainan University, Haikou 570228, China; 2 National Engineering Research Center for the Emergency Drug, Beijing Institute of Pharmacology and Toxicology, Beijing 100850, China; 3 Xishuangbanna Tropical Botanical Garden, Chinese Academy of Sciences, Xishuangbanna 666303, China; 4 Southeast Asia Biodiversity Research Institute, Chinese Academy of Sciences & Center for Integrative Conservation, Xishuangbanna Tropical Botanical Garden, Chinese Academy of Sciences, Xishuangbanna 666303, China

**Keywords:** *Zanthoxylum acanthopodium* DC., ethnobotany, essential oil, dengue virus, *Aedes albopictus*, larvicide

## Abstract

*Zanthoxylum acanthopodium* DC. is a widely used traditional medicinal plant to treat fever, flu, stomachache, traumatic injury, and mosquito bite in tropical and subtropical Asia. This study aimed to investigate the antifungal, anti-inflammatory, antiviral, and larvicidal activities of its fruit essential oil. The essential oil sample from China (EOZC) was mainly composed of limonene (29.78%) and β-myrcene (26.65%), while the sample from Myanmar (EOZM) was dominated by Terpinen-4-ol (43.35%). Both essential oils showed antifungal activity, with 90% minimum inhibitory concentration (MIC_90_) values ranging from 26.3 to 499 μg/mL. By obviously inhibiting nitric oxide (NO) in RAW 264.7 cells, EOZC (IC_50_, 16 μg/mL) showed comparable anti-inflammatory activity to the positive control L-NMMA (IC_50_, 12.2 μg/mL). EOZM showed significant antiviral activity against the dengue virus with an IC_50_ value of 13 μg/mL. Additionally, both EOZC and EOZM demonstrated dose-dependent larvicidal activity against *Aedes albopictus*, with LC_50_ and LC_90_ values ranging from 45.8 to 144.0 μg/mL. Our results contribute a theoretical foundation for the further application of *Zanthoxylum acanthopodium* DC. as an antifungal and anti-inflammatory ingredient in the pharmaceutical industry and further indicate that it has the potential to be developed as a new source of natural and eco-friendly medicine for the prevention and treatment of dengue virus.

## 1. Introduction

The genus *Zanthoxylum* (Rutaceae), distributed mainly in tropical and subtropical regions of Asia, Africa, Oceania, and North America, consists of approximately 250 species, of which 39 species and 14 varieties were found in China [1]. Fruits from this genus are commonly used as spices, antiseptics, and insecticides worldwide. Earlier studies have shown that *Zanthoxylum* plants have antioxidant, antibacterial, insecticidal, immunomodulatory, anti-malignant cell proliferation, anti-inflammatory, and analgesic properties [2,3,4,5,6,7]. *Zanthoxylum acanthopodium* DC., mainly distributed in China, Myanmar, India, and Nepal, is used as a medicine to cure many diseases and as a spice in cooking by local people [1]. Ethnobotanical research revealed that it was called “ge ma ga”, “mo zi la”, or “za bu ga biu” by local Dai, Hani, and Yao people, and its roots and fruit were used to treat cold, stomach pain, bruising and injury in Yunnan and Guizhou, China [8,9,10,11,12,13]. In Myanmar, local Chin people name it “Dan bung hling” and use its crushed fruits to cure toothache, mosquito, and leech bites, or boil its fruits and leaves and then apply them as warm baths [14]. In northern Sumatra, Indonesia, it was named “andaliman” and used as a spice to prepare traditional fish dishes [15]. In India, it is also used as a spice and wild vegetable and its fruit is rich in fatty acids, and the leaves have high contents of protein, potassium, calcium, and phenolic compounds [16,17,18]. Its methanol, ether, and ethanol extracts showed antibacterial, antioxidant, and anti-inflammatory activities [19]. Its stem essential oil from Malaysia expressed larvicidal activity against *Aedes aegypti* larvae [20]. Ethanol extraction of its fruits presented anticancer activities [21]. It was reported that the essential oil extracted from the aerial part of *Z. acanthopodium* collected in May was rich in estragole (15.46%) and eucalyptol (10.94%) and showed larvicidal activity against the malaria mosquitoes *Anopheles anthropophagus* and *Anopheles sinensis* [22]. In addition to the explorations mentioned above, few studies have focused on the chemical composition and biological activity of the fruit essential oils of *Z. acanthopodium* collected from different countries.

Dengue fever is a predominantly viral disease by *Aedes albopictus* and *Aedes aegypti*. In 2019, World Health Orgnization (WHO) listed dengue fever as one of the top 10 global public health threats, noting that it can cause the death of 20% of severe cases [23]. According to the WHO report, the global incidence of dengue fever has increased eight-fold in the past two decades and there are approximately 1–4 million infection cases every year [24]. Nearly 70% of those at high risk of being infected are in the Asia-Pacific region [25]. Moreover, global climate change could exacerbate the crisis, and dengue outbreaks are likely to occur more frequently and spread further, even to higher elevations [26]. Recent research suggests that urbanization will drive dengue’s global spread and estimates that by 2080, dengue fever will threaten 6 billion people in 197 countries or 60 percent of the world’s population [27]. The global outbreak of dengue fever has strained public immunization systems and caused substantial economic losses. There are four different subtypes of dengue fever. A globally approved dengue vaccine is still under development, and there is no specific treatment for dengue fever [28]. In addition, studies have shown that residents in China and Southeast Asia also face potential threats from the Zika virus [29].

Recent studies found that essential oils demonstrated anti-mosquito and mosquito repellent effects [30,31,32,33]. Essential oil is a mixture of several compounds collected from aromatic plants and has been frequently used in pharmaceuticals, preservatives, households, and cosmetic products since ancient times [34]. Compared with synthetic insecticides, essential oils are likely to cause less pollution to the environment and less toxic to humans [35]. The mechanism by which essential oil is lethal to mosquitoes is complicated. Thus, it is difficult for mosquitoes to develop resistance to essential oils [36,37]. Without an effective vaccine, the best way to control dengue fever is to limit the population of mosquitoes [28,38,39]. Therefore, it is of great significance to develop eco-friendly insecticides from essential oils to eliminate mosquitoes.

To verify its traditional use and provide a scientific basis for the sustainable use and commercial development of *Z. acanthopodium* in the pharmaceutical field, we extracted its essential oils from fruits collected from China and Myanmar and investigated their antifungal, anti-inflammatory, antiviral, and larvicidal activities.

## 2. Results and Discussion

### 2.1. Essential Oil Composition

The essential oil yields were 2.62% and 4% (*w*/*w*) on a dry weight basis for *Z. acanthopodium* fruits from China (EOZC) and the sample from Myanmar (EOZM) respectively. GC/MS analyzed both essential oils, and their chemical compositions are shown in Table 1. There were 36 chemical components identified in the two samples, and the identification rates of EOZC and EOZM were 96.96% and 98.17%, respectively. Both essential oils had 17 chemical components in common, while the content of their major components varied widely. Specifically, the EOZC was mainly composed of limonene (29.78%) and β-myrcene (26.65%), while the EOZM was dominated by terpinen-4-ol (43.35%). The steam distillation extracted essential oils of *Z. acanthopodium* fruits from Medan, Indonesia, had 29 chemical compositions with geranyl acetate (23.18%), citronella (11.23%), and β-citronelol (10.64%) as the main chemical components and had different influences in terms of locomotor activity dependent on doses given to mice [40]. The n-hexane extracted essential oils of *Z. acanthopodium* fruits from Sumatra Island, Indonesia, had 32 chemical compositions, with carveol (47.70%) and myrtenyl acetate (12.55%) as the dominant components and expressed antidiabetic activity [41]. Steam distillation extracted essential oils of *Z. acanthopodium* fruits from Meghalaya state, India, had 21 chemical compositions, with eucalyptol (36.56%), limonene (16.90%), and δ-3-carene (13.53%) as the primary components and showed promising antibacterial activity against *Staphylococcus aureus* [42]. Thus, we found that the essential oils of *Z. acanthopodium* fruits, collected from different geographical sites and extracted by different methods, had significant differences in their major components and biological activities, indicating that the exploration of the essential oil composition of the same species collected from different regions remains both very important and enlightening.

### 2.2. Antifungal Activity

Two essential oils displayed significant activity against four tested fungi (Table 2). EOZC showed the best antifungal activity against *Epidermophyton floccosum*, with 90% minimun inhibitory concentration (MIC_90_) values of 26.3 μg/mL. Generally, EOZC exhibited better antifungal activity than EOZM. The antifungal activity of EOZC may be due to its higher content of limonene, which has shown significant antimicrobial activity [43]. The results showed that essential oils had sound inhibitory effects on the four tested fungi, which could cause skin infections and itching, and may partly explain why local people like to boil the fruit of *Z. acanthopodium* for bathing.

### 2.3. Anti-Inflammatory Activity

**Figure 1:**
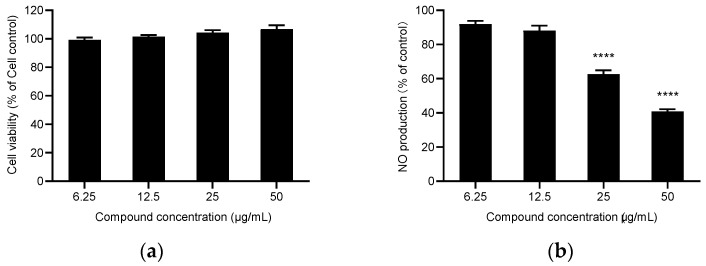
Anti-inflammatory activity of the essential oil of Z. acanthopodium fruits from Myanmar (EOZM). (**a**) The cytotoxicity of EOZM in RAW 264.7 cells. Cell viability were by MTS assay. (**b**) The inhibitin of EOZM on LPS-induced NO production in RAW 264.7 cells. Results were presented as mean ± standard deviation (SD) of three independent tests, **** *p* < 0.0001.

**Figure 2:**
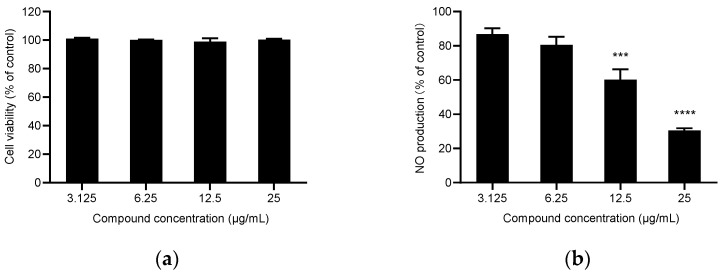
Anti-inflammatory activity of the essential oil of Z. acanthopodium fruits from Myanmar (EOZC). (**a**) The cytotoxicity of EOZC in RAW 264.7 cells. Cell viability were by MTS assay. (**b**) The inhibitin of EOZC on LPS-induced NO produc-tion in RAW 264.7 cells. Results were presented as mean ± standard deviation (SD) of three independent tests, *** *p* = 0.002, **** *p* < 0.0001.

Nitric oxide (NO) has an essential role in inflammation, tumors, and the cardiovascular system [44]. Therefore, the inhibition of NO production is a direct indicator of the compound’s anti-inflammatory activity. At a concentration without an effect on the viability of the cells (Figure 1 and Figure 2), the results showed that the two essential oils had significant NO inhibitory activity (Table 3). EOZC showed better anti-inflammatory activity with IC_50_ values of 16 μg/mL, similar to the positive control drug L-NMMA, with IC_50_ values of 12.2 μg/mL. The anti-inflammatory activity of the two essential oils might be attributed to their high contents of limonene, β-myrcene, and terpinen-4-ol, which all demonstrated apparent anti-inflammatory activity [45,46,47].

NO is one of the critical determinants in infection and inflammation, so the significant NO inhibition effect supports the traditional use of Z. acanthopodium fruits as a remedy for inflammation-related diseases, such as toothache and stomachache, and swelling and pain caused by injury or mosquito and leech bites.

### 2.4. Larvicidal Activity

**Figure 3:**
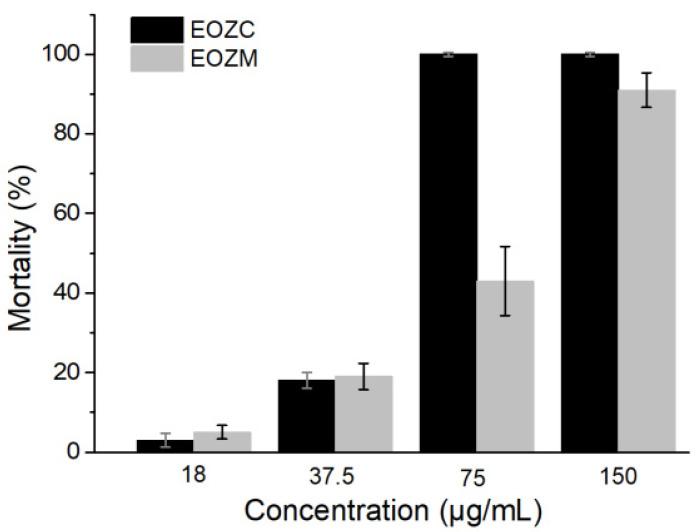
Larvicidal activity of essential oils from Z. acanthopodium fruits.

The essential oil from Myanmar or China expressed obvious larvicidal activity against Aedes albopictus larvae in a dose-dependent manner at concentrations of 18–150 μg/mL (Figure 3). The LC_50_ values of EOZC and EOZM were 45.8 μg/mL and 87 μg/mL, respectively. The LC_90_ values of EOZC and EOZM were 59.4 μg/mL and 144.0 μg/mL, respectively. Their larvicidal activities were lower than the positive control chlorpyrifos, with LC_50_/LC_90_ values of 0.0037 and 0.0064 μg/mL, respectively.

The variation in the main components of essential oils resulted in different lethal rates for Aedes albopictus larvae. On the one hand, limonene, β-myrcene, and terpinen-4-ol have been reported to have larvicidal activity against various mosquitoes, including Aedes albopictus. On the other hand, limonene and β-myrcene showed better larvicidal activity than terpinen-4-ol in previous reports [48]. Thus, EOZC, which had much higher contents of limonene and β-myrcene, showed more vigorous larvicidal activity than EOZM. Aedes albopictus is one of the main vectors for transmitting dengue fever and the Zika virus [49]. Due to the lack of effective vaccines for dengue fever, the primary method to control or prevent dengue virus transmission is to combat mosquito vectors [24]. Our result could be a positive example of the traditional usage of Zanthoxylum plants as insecticides. Furthermore, EOZC and EOZM showed good larvicidal activity against Aedes albopictus larvae at relatively low concentrations, indicating that Z. acanthopodium fruits have the potential to be developed as a novel and natural agent for the control of mosquitoes.

### 2.5. Antiviral Activity

Two essential oil samples were active against the dengue virus, while only EOZM showed an inhibitory effect against the Zika virus with an IC_50_ value of 94 μg/mL (Table 4). EOZM showed significant inhibition of DENV with an IC_50_ value of 13 μg/mL, which was approximately 5-fold lower than that of the positive control (NIT008). The selective index (SI) of EOZM against DENV was 14, indicating a significant safety range [50]. Although scientists in many countries have made many efforts and discovered a variety of plant extracts that have inhibitory ability against the dengue virus [51,52], specific medicines to cure dengue fever are still being explored. Our findings might provide a new natural and eco-friendly agent for developing highly effective medicine to inhibit the dengue virus. As the mechanism for essential oils’ larvicidal and antiviral activity is detailed, further studies are needed to apply this plant better.

## 3. Materials and Methods

### 3.1. Chemicals and Reagents

Dimethylsulfoxide (DMSO), fetal bovine serum (FBS), penicillin–streptomycin, L-glutamine, and lipopolysaccharide (LPS) were purchased from Sigma–Aldrich (St. Louis, MO, USA). Dulbecco’s modified Eagle’s medium (DMEM) was purchased from Thermo Scientific (Logan, UT, USA). CellTiter 96^®^ AQueous One Solution Reagent for the MTS assay and the Griess reagent system for NO measurement were purchased from Promega Corporation and purchased from Promega Corporation (Madison, WI, USA). Ceftazidime was purchased from CAS mart. Penicillin G Na was purchased from Biosharp company. Terbinafine hydrochloride and amphotericin B were purchased from Sigma Company. Candida albicans (ATCC 10231) was purchased from the American Microbiologics Company. Epidermophyton floccosum (CBS 566.94), Trichophyton rubrum (ATCC 4438), and Microsporum gypseum (CBS 118893) were purchased from the China Center for Medical Culture Collection (CMCC). Standard Mueller–Hinton agar and broth (MHA and MHB) and Sabouraud agar and broth (SA and SB) were purchased from Huankai Microbial Technology Company (Guangdong, China). All reagents were analytical standards.

### 3.2. Plant Materials and Essential Oil Extraction

With the help of local villagers, *Z. acanthopodium* fruit samples were collected from Nat ma Taung National Park, Chin State, Myanmar, and Ailao Mountain, Yunnan, China, in September 2019 and were identified by Mr. Shi-shun Zhou from Xishuangbanna Tropical Botanical Garden, Chinese Academy of Sciences. A voucher specimen (no. 135658) was deposited in the herbarium (HITBC). The essential oils were extracted following the reproted procedure with minor modification [34]. Briefly, the samples (100 g), which were dried in shade and ground with a laboratory mill, were extracted with 2000 mL boiling water in a Liens-Nickerson simultaneous steam distillation continuous extraction (SDE) with 30 mL ether as the solvent for 3 h. The essential oil and solvent were collected together, and then ether was removed using a rotary evaporator to yield the essential oil at room temperature and room pressure. The collected essential oils were stored at −18 °C in the dark for further testing.

### 3.3. Analysis of Chemical Composition

Essential oils were characterized and quantified by GC-MS analysis on an Agilent 7890 gas chromatograph (GC) instrument equipped with an Agilent 5975 mass spectrometer (MS) and an HP-5MS capillary column (30 m × 250 μm × 0.25 μm, 5% Pheny Methyl Silox). The loading gas was helium at a 1.0 mL/min current flow rate. The temperature program for the oven is as follows: 40 °C for 1 min, then 40 °C to 150 °C at 3 °C/min, and increased to 250 °C at 10 °C/min, then hold for 10 min, the total running time was 57.667 min. The components were identified by comparing calculated experiment GC retention indices, which were determined concerning homologous series of n-alkanes C7-C30 under identical experimental conditions, with the GC retention indices reported in NIST Standard Reference Database (NIST Chemistry Web Book, 2014), by matching their mass spectra with those recorded in the NIST 08 database (National Institute of Standards and Technology, Gaithersburg, MD, USA) and mass spectra with published data. The relative percentage composition of individual components of the essential oils was calculated according to the GC peak area by normalization without the use of correction factors.

### 3.4. Antifungal Activity

#### 3.4.1. Microbial Strains and Culture Media

*Candida albicans* (ATCC 10231) was purchased from Microbiologics; *Epidermophyton floccosum* (CBS 566.94), *Trichophyton rubrum* (ATCC 4438), and *Microsporum gypseum* (CBS 118893) were purchased from the China Center for Medical Culture Collection (CMCC). Sabouraud agar and broth (SA and SB) were used as the fungal culture media.

#### 3.4.2. Antifungal Test

*Candida albicans*, *Microsporum gypseum*, *Epidermophyton floccosum,* and *Trichophyton rubrum* were tested for susceptibility. *Candida albicans* was prepared to a final concentration of 1 × 10^5^ CFU/mL, and the others were prepared to a final concentration of 5 × 10^5^ CFU/mL. They were incubated in different concentrations of both essential oils in 96-well plates. Candida albicans were incubated at 37 °C for 24 h, and the others were incubated at 25 °C for 5 day. Amphotericin B was the positive control drug for *Candida albicans*, and terbinafine hydrochloride was the positive control drug for other types of fungi. Media without antimicrobial agents were used as negative controls, and sterile culture medium without drugs and fungi was used as a blank control. The absorbance was measured at 625 nm using a microplate reader. The MIC_90_ was calculated by Reed & Muench [53].

### 3.5. Anti-Inflammatory Activity

#### 3.5.1. Cell Culture

The murine macrophage cell line RAW 264.7 was obtained from the Kunming Institute of Zoology, Chinese Academy of Sciences (KCB200603YJ), maintained in DMEM containing 10% fetal bovine serum, 1% penicillin-streptomycin, and 1% L-glutamine at 37 °C in a 5% CO_2_ incubator and incubated every 2 day.

#### 3.5.2. Cell Viability Assay

As previously reported, cell viability was evaluated by MTS assay [54]. In the MTS assay, 100 µL cell suspensions (1.5 × 10^5^ cells/well) were cultivated in 96-cell plates for 18 h, as described. Then, the cells were conditioned with different concentrations of essential oils for 30 min and incubated for an additional 24 h with 1 μg/mL LPS. Finally, 20 µL of CellTiter 96^®^ AQueous One Solution Reagent, prepared by MTS in the presence of phenazine ethosulfate (PES), was added to each well and incubated for 1 h at 37 °C in a 5% CO_2_ incubator. A multifunctional microplate reader measured each well’s absorbance at 490 nm. The results are expressed as a percentage of MTS production by control cells maintained in a culture medium. A range of concentrations with no effect on cell viability was selected to test the inhibitory activity against NO.

#### 3.5.3. Measurement of NO Production

NO production in LPS-stimulated RAW 264.7 cells was measured by nitrite accumulation in the culture supernatant using the Griess reagent system according to the manufacturer’s directions. Cells were inoculated at 1.5 × 10^5^ cells/well in 96-well culture plates and then incubated with medium (control) for 18 h. The cells were pretreated with different concentrations of essential oils for 30 min, stimulated with 1 µg/mL LPS, and cultured for 24 h. Briefly, 50 µL of culture supernatants were collected and mixed with the Griess reagent system and then incubated in the shade for 10 min. A multifunctional microplate reader measured each well’s absorbance at 570 nm. L-NMMA, a well-known nitric oxide synthase (NOS) inhibitor, was used as a positive control, and untreated cells incubated in culture medium were employed as blank control. The nitrite concentration was determined from a sodium nitrite standard curve.

### 3.6. Larvicidal Activity

The eggs of *Aedes albopictus* were provided by the Centers for Disease Control of Yunnan Province, China. The mosquitoes were reared at 27 ± 2 °C and 70 ± 10% relative humidity with a 14 h light and 10 h dark photoperiod. Both essential oils were tested for larvicidal activity based on the recommendations of the WHO with slight modifications [55]. Briefly, 25 mosquito larvae of 4th instar were introduced with 99 mL dechlorinated water into the beaker. The essential oils were dissolved in 1 mL ethanol and prepared at different concentrations. The chemical larvicide chlorpyrifos was used as a positive control, and solvent ethanol served as a blank control. During the test, no food was supplied to the larvae. All the tests were replicated four times, and larval mortality was calculated after the 24 h exposure period.

### 3.7. Antiviral Activity

Antiviral activity was evaluated for Zika and dengue viruses in BHK cells. BHK cells were seeded into 96-well culture plates at 5 × 10^3^ cells/well and cultured overnight. Both essential oils were gradient diluted using 2% DMEM and added to a 96-well culture plate. Then, BHK cells were inoculated with 100 TCID_50_ of Zika or dengue virus. In this assay, NITD008 was used as a positive control. At 8 days post-infection (dpi), cell viability was evaluated by CellTiter-Glo^®^ Luminescent Cell Viability Assay following the manufacturer’s instructions. The results were expressed as the 50% inhibitory concentration (IC_50_) and 50% cytotoxic concentration (CC_50_).

### 3.8. Statistical Analysis

All experiments were performed at least three times and expressed as the mean values ± standard deviation (SD). The 50% inhibition concentration (IC_50_) was calculated by probit regression analysis using SPSS 17.0 for Windows (SPSS Inc. Chicago, IL, USA). One-way ANOVA with Dunnett’s multiple comparison test and least significant difference (LSD) test were performed using SPSS 17.0 for statistical evaluation. Differences were accepted as significant at *p* < 0.05.

## 4. Conclusions

The results showed that the essential oils of *Z. acanthopodium* fruits collected from China and Myanmar were rich in limonene, β-myrcene, and terpinen-4-ol, had suitable inhibitory activities against four kinds of human epidermal fungi, and showed prominent anti-inflammatory activities by significantly inhibiting NO content in RAW264.7 cells. Our results verified its traditional medicinal value and supported its further development in the pharmaceutical industry. For the first time, our results demonstrated that *Z. acanthopodium* fruit essential oil could not only eliminate *Aedes albopictus* larvae, but also inhibit dengue virus at relatively low concentrations, indicating that it has the potential to be developed as a new and natural medicine to control or prevent the transmission of dengue virus.

## Figures and Tables

**Table 1:** Chemical composition of essential oils from *Z. acanthopodium* fruits.

No	RT ^1^	RI^cal 2^	RI^lit 3^	Component	EOZM (%)	EOZC (%)
1.	9.22	925	926	α-Thujene	0.65	0.14
2.	9.47	931	930	α-Pinene	0.62	4.18
3.	10.06	945	945	Camphene	- ^4^	0.29
4.	11.16	971	972	Sabinene	3.34	6.78
5.	11.25	973	974	β-Pinene	0.39	5.80
6.	12.01	992	992	β-Myrcene	1.48	26.65
7.	12.52	1003	1004	α-Phellandrene	0.68	1.25
8.	13.09	1016	1018	(+)-4-Carene	5.65	0.49
9.	13.46	1024	1025	o-Cymene	1.70	0.11
10.	13.67	1028	1028	Limonene	8.71	29.78
11.	13.72	1029	1031	Eucalyptol	9.03	-
12.	14.64	1049	1050	β-Ocimene	6.11	7.18
13.	15.07	1058	1058	γ-Terpinene	8.44	0.78
14.	15.45	1066	1161	(*E*)-β-terpineol	0.24	0.37
15.	16.43	1088	1088	Terpinolene	2.00	0.45
16.	17.07	1101	1101	Linalool	0.17	0.46
17.	17.25	1105	1105	Nonanal	-	0.29
18.	17.99	1121	1120	(*E*)-p-2-Menthen-1-ol	1.21	0.24
19.	19.09	1144	1145	Verbenol	-	0.22
20.	20.06	1165	1164	Borneol	-	0.22
21.	20.61	1177	1177	Terpinen-4-ol	43.35	1.56
22.	21.26	1191	1192	α-Terpineol	3.11	0.42
23.	21.46	1195	1195	(*Z*)-Piperitol	0.24	-
24.	21.51	1196	1196	(-)-Myrtenol	-	1.62
25.	22.06	1208	1206	(*E*)-Piperitol	0.39	-
26.	25.58	1286	1285	Bornyl acetate	-	0.90
27.	26.23	1300	1297	(*E*)-Pinocarvyl acetate	-	0.34
28.	27.34	1326	1327	Myrtenyl acetate	-	0.65
29.	28.39	1350	1350	α-Terpinyl acetate	0.66	-
30.	31.31	1419	1419	Caryophyllene	-	1.46
31.	32.72	1453	1455	Humulene	-	0.25
32.	33.86	1481	1481	Germacrene D	-	0.34
33.	35.58	1525	1525	(+)-δ-Cadinene	-	0.70
34.	39.66	1650	1647	τ-Muurolol	-	1.08
35.	39.78	1654	1648	τ-Muurolol	-	0.28
36.	39.98	1662	1658	α-Cadinol	-	1.69
Total identified					98.17	96.96

^1^ Retention time. ^2^ The retention index experimentally calculated using C_7_-C_30_ alkanes. ^3^ The retention index taken from NIST database. ^4^ Not detected.

**Table 2:** Antifungal activity of essential oils from *Z. acanthopodium* fruits.

Treatment	MIC_90_ (μg/mL) ^1^			
	*Candida albicans*	*Epidermophyton floccosum*	*Trichophyton rubrum*	*Microsporum gypseum*
Amphotericin B	0.06 ± 0.001	- ^2^	-	-
Terbinafine Hydrochloride	-	0.02 ± 0.001	2.2 ± 0.16	0.01 ± 0.001
EOZM	-	95 ± 3.3	506 ± 3.3	372 ± 3.7
EOZC	499 ± 2.2	26.3 ± 0.32	91 ± 2.1	82.6 ± 0.61

^1^ 90% minimun inhibitory concentration. ^2^ Not determined.

**Table 3:** Anti-inflammatory activity of essential oils from Z. acanthopodium fruits.

Compound	IC_50_ ^1^ (μg/mL)
L-NMMA	12.2 ± 0.65
EOZM	37 ± 2.0
EOZC	16 ± 1.6

^1^ 50% inhibitbitory concentration.

**Table 4:** Antiviral activity of essential oil from Z. acanthopodium fruits.

Compound	ZIKV ^1^ (μg/mL)			DENV ^2^ (μg/mL)		
	IC_50_^3^	CC_50_^4^	SI ^5^	IC_50_	CC_50_	SI
EOZM	94 ± 19.8	372 ± 103.8	3.9 ± 0.28	13 ± 1.4	183 ± 92.1	14 ± 5.7
EOZC	- ^6^	>800	-	184.86 ± 64.49	>800	>4.35
NITD008	0.4 ± 0.01	>3	>8.13	2.5 ± 0.01	>3	>1.16

^1^ Zika virus. ^2^ Dengue virus. ^3^ 50% inhibitory concentration. ^4^ CC_50_ means 50% cytotoxic concentration. ^5^ selective index. ^6^ not determined.

## Sample Availability

The samples from *Z. acanthopodium* fruits are available from the authors.
